# Neuralgia

**Published:** 1872-12

**Authors:** Walter Hay

**Affiliations:** Assistant to Chair of Practical Medicine, and Lecturer on Diseases of Brain and Nervous System, Rush Medical College; Chicago


ARTICLE IV.—LECTURE 2ND —*Neuralgia.* By WALTER HAY, M.D., Assistant to Chair of Practical Medicine, and Lecturer on Diseases of Brain and Nervous System, Rush Medical College.

GENTLEMEN: Having in my last lecture indicated to you the prominent outlines of peripheral anæsthesia, I will now proceed to direct your attention to the opposite pathological state of the nervous system, hyperæsthesia, or as more generally called, neuralgia, implying nerve-pain.

The term hyperæsthesia, applied to this condition by Romberg, is, like most of our technology, of Greek origin, and is defined by that author as "exalted irritability and increased irritation of the sensitive or centripetal nerves."

Anstie, one of the most recent, as well as one of the best writers upon the subject, objects to the term hyperæsthesia, upon the ground that it implies exaltation of function, and the term, or idea of function, being normal, cannot be applied to pain, which is abnormal. His language is as follows:

"1st. Pain is not a true hyperæsthesia; on the contrary, it involves a lowering of true function.

"2nd. Pain is due to a perturbation of nerve-force, originating in dynamic disturbance either within or without the nervous system.

"3rd. The susceptibility to this perturbation is great in proportion to the physical imperfection of the nervous tissue, *until* this imperfection reaches to the extent of cutting off nervous communication (paralysis)."

This is in reality a mere battle of words against a phantom of the author's own creation. His ultimate object, however, is to impress upon his readers the fact that pain, in general, and neuralgic pain in particular, is an expression of a debilitated condition of the system.

Neuralgia, then, may be defined as a disease of the nerves characterized by acute darting, lancinating, boring or burning pains, following the course of sensory or afferent nerves, intermittent in their duration, and usually unilateral, unaccompanied by febrile or inflammatory reaction.

This description will cover the vast majority of cases; in some, however, the pains are continuous or aching, but these will be referred to later.

The intermissions are usually complete, but not always, and generally, although not invariably, regular, that is, recurring after regular intervals; during these intervals the patient feels perfectly well, indeed the transition from intense suffering to complete relief is very striking.

This class of diseases was first described by Aretæus, but we find no very distinct description of it again, until that of Andre, whose account of the malady was published in Paris in 1756.

Fothergill, a little later, 1782, published in London his treatise upon painful affections of the face, containing a very good description of facial neuralgia. In 1782 a memoir was presented to the Royal Society of Medicine, Paris, by Thouret, in which a form of facial neuralgia is described, to which the name "tic douloureux" was first applied.

The different varieties of facial neuralgia were first classified by Chaussier in a memoir published in Paris in the year XI of the French Revolution, or 1802. Reverdit, a French author, likewise published in Paris in 1817, an inaugural thesis entitled "A Dissertation on Facial Neuralgia or Prosopalgia," in which pain upon pressure is first indicated as a symptom of the disease. In the same year,

1817, a thesis upon the same subject was also published by Barbarin. An excellent treatise (for that date) upon facial neuralgia was that of I. Frank, of Turin, published in 1822.

Regnier, of Paris, wrote upon the same disease in 1819. Chaponniere in 1732 wrote a treatise upon the seat and causes of facial neuralgia; and in 1834 the intermittent character of the disease was specially noted by Bellingeri, in a memoir published in Turin, from observations made upon 5,612 cases. This view (of the intermittent character of neuralgia) was sustained by Rennes in 1836; and in the same year Brerard limited facial neuralgia to the fifth pair of nerves; although two years later, in 1838, Jobert de Lamballe controverted this proposition, asserting that all the nerves of the face were subject to this disease.

In 1840, the Sydenham Society, of London, published a translation, by Dr. Sieveking, of the Manual of Diseases of the Nervous System of Man, by Moritz H. Romberg, Professor of Medicine at the University of Berlin, and this great work, together with that of Valleix, "Treatise on Neuralgias and Painful Affections of the Nerves," published in Paris in the following year, 1841, constitute the sources from which the most that is now known and written upon the subject is drawn.

Of cotemporary writers, Charles Bland Radcliffe, C. Handfield Jones, and Anstie, of London, and Hammond, of New York, have enriched the literature of the subject, and condensed into available form the gist of all which is worth knowing thereupon. The best and most comprehensive summary is that of Anstie, and I would advise those of you who desire to familiarize yourselves with the disease to study his little work.

Neuralgias may be classified, as to their origin, into—

1st. Malarial, or those occasioned by exposure to miasmatic influence, and these are nearly related to intermittent fever or ague, in which the effect of malarial influence is manifested through the motor, instead of, as in the case under discussion, through the sensory, nerves.

2nd. Neuralgias of the period of developmental activity, *i. e.*, in early life, in which nerve tissue in common with all others may be supposed to be in a condition of instability, by reason of the rapid nutritive changes which it sustains at this time, and hence extremely liable to functional perversion.

3rd. Neuralgias of the middle period of life, when the wear and tear of brain and nerve is at its maximum.

4th. Those of the period of decay, when the condition of nerve tissue already referred to as incidental to the developmental period of life, has been re-established, when by reason of the disturbance in the equilibrium between nutrition and decay, between advancing and retrograde metamorphosis of tissue, a state of organic instability again occurs—a state corresponding to Romberg's definition, "exalted irritability."

5th. And lastly, neuralgias of anæmia or mal-nutrition, in which again the condition of nerve tissue is one of instability, for the same reason as has been assigned in the last preceding class of cases.

Throughout the classification you will perceive that there is one factor common to every class, and that factor is an enfeebled condition of nerve power, debility, an unstable state of nerve tissue, increased mobility of its constituent molecules, in consequence of which its capacity to resist the influence of disturbing causes is diminished. A full appreciation of this fact will materially aid you in the treatment of disease in your future career.

The causes of neuralgia will comprehend the influences just referred to, and, in addition, cold externally applied—as for example, a draught of air, and there is perhaps no more frequent cause; certain poisons, such as lead and syphilis; the rheumatic and gouty diathesis. With regard to the influence of lead in producing neuralgia, certain authorities deny the neuralgic character of lead colic, although the careful observation of a few cases of this disease will, I think, demonstrate most completely, that it belongs to the category of true neuralgias. The same authority who denies the neuralgic character of lead colic, admits the existence of lead palsy; it would seem no more than consistent to infer corresponding effects upon the different tracts of the same nerve from the operation of a common cause.

Neuralgias may be still further classified topographically into superficial and visceral; the first of these comprises those involving the superficial nerves, and the other, the nerves supplying the various internal organs.

Of superficial neuralgias the most important are:

1st. Those of the fifth nerve, and its various subdivisions.

2nd. Cervico-occipital neuralgias, involving the first four pairs of spinal nerves, that is to say, the posterior roots and ramifications of the first four pairs.

3rd. Cervico-brachial.

4th. Intercostal.

5th. Lumbo-abdominal.

6th. Crural.

7th. Sciatic.

The neuralgias of the fifth pair constitute the most important group. The exposed situation of the branches of this nerve, ramifying as it does over the face, seems to render it peculiarly obnoxious to some of the most frequent causes of this disease, and its intricate connections with the various cephalic ganglia of the sympathetic subject it to the influence of all the changes incident to digestive and nutritive functions.

Of the neuralgias of the fifth pair, those of the first or ophthalmic division are perhaps the most important and common, and I think the most troublesome. They may be designated according to the nerves affected, as supra-orbital, trochlear, palpebral, nasal, and ocular, and these as well as all others appear to have their origin in certain foci; these foci of pain are the points at which the nerves emerge from the bones and approach the surface. In the first of these the pain is located over the eyebrow, involving the supra-orbital branches of the ophthalmic division of the fifth pair. In the nasal, the pain is perceived at the point of emergence of the nasal nerve from the bone near the junction of the bone with the cartilage. Tenderness to the touch at these foci, and at other points along the course of the affected nerve, and great increase in the pain resulting from pressure upon these points, have been insisted upon by Valleix, as diagnostic of true neuralgias. This is disputed by other writers, who deny the existence of the tenderness. This, gentlemen, is one of the instances of the "disagreement of doctors." They are both right and both wrong, in accordance with the period of the disease at which the observation was made. My own experience with neuralgia has been somewhat extensive, and in the highest degree practical, and I therefore speak with less hesitation. These painful points, "points douloureuses" of Valleix, are not usually to be detected in recent cases of true neuralgia. In long standing cases, however, it is quite common to find tenderness at certain points along the course of the affected nerve, more especially at the points of emergence of the nerve from the bone, above referred to. There is good reason, however, to believe that this tenderness is due to a low degree of inflammatory action in the tissues contiguous to the nerve, as it usually persists for a certain length of time after the cessation of the neuralgic pain. In recent cases it is not uncommon to find that pressure firmly applied will diminish the painful sensation, apparently by intercepting the transmission of morbid, or perverted, sensations to the sensorium. Of the neuralgias of this, the first or ophthalmic division of the fifth pair of nerves, the most important is that termed, by Anstie and others, trochlear, and by Valleix and the older French authorities, migraine, hemicrania, and by its unfortunate victims, sick headache.

I will give you a description of this fearful malady, drawn from a large number of clinical observations, and which you will find to be so accurate, that should you ever suffer from an attack, you will recognize it from the description. The attack usually begins in the early morning, the patient being awakened from sleep by an uneasy sensation about the head, sometimes not amounting to pain, and sometimes even a slight dull pain at the trochlea or groove upon the inner portion of the superciliary ridge. A little later he is attacked by rigors, coldness of the extremities, and of the whole surface, the fingers appearing shrivelled and shrunken at their extremities. There is at this time frequent and uncontrollable yawning or gaping; the countenance becomes pale and shrunken; the eyes seem to sink into their orbits; the conjunctivæ become pale and the pupils contracted. The pulse becomes contracted and wiry, and the respiration sighing. The pain has by this time increased to a frightful degree of severity, radiating from its initial point above referred to, over the corresponding half of the forehead, and extending about half the distance from the frontal to the parietal protuberance, but never passing over these limits upon one side. Unlike most forms of neuralgia, hemicrania is occasionally double, affecting both sides of the head alike, although never commencing in both at the same time, the second side becoming involved after the disease has reached its maximum of intensity upon the other. Sometimes, moreover, the disease migrates from one side to the other, ceasing altogether in the side first attacked, soon after its commencement in the second.

The disease is accompanied throughout its course by more or less nausea, with great distress in the epigastrium, frequently by retching, and often by vomiting, which may continue with great violence for ten or twelve hours.

As these attacks may be often preceded by some imprudence in diet, it has been supposed, not unreasonably perhaps, that they are caused by dyspepsia, and hence the hope of relief by means of the vomiting, is indulged in; but the hope is illusory; vomiting brings no relief, indeed in many cases appears to aggravate the pain. In truth, dyspepsia is not the cause, but only an associated result with the neuralgia of a common cause, nervous exhaustion.

Exhaustion of vaso-motor power, is an extremely frequent precursor of this form of neuralgia, and may be itself the result of over fatigue, either mental or bodily.

The second or superior maxillary division of the fifth pair of nerves, has also its foci of pain, corresponding exactly with the points of emergence of the various branches of this division, from their bony canals, viz.: the infra-orbital, the malar, the superior labial, and the palatine. The latter is rare; I have seen but one case, which was, however, of great intensity.

So of the inferior maxillary division; there are painful points, upon the inferior dental nerve, the lingual and the inferior labial.

Some authors speak of a focus of pain a little above the parietal eminence, as the most common of all in this division. I have observed it, but not so frequently, as many others.

I mentioned above that vaso-motor exhaustion was a most common precursor of this form of disease. The immediate effect of this is dilatation of the vessels supplying the nervous centres, and at the same time contraction of the superficial vessels, indicated by coldness of the surface.

The termination of these attacks is usually marked by sudden perspiration, and a copious secretion of limpid urine.

The second group of neuralgias to which I have referred, as already mentioned, involve the first four pairs of spinal nerves, *i. e.*, their posterior roots, and are perceived in the triangular space included between the margin of the lower jaw, the sterno-cleido mastoid muscle, and the median line of the neck, also in the parotid region and space behind the ear.

The cervico-brachial neuralgias have been mapped out topographically by Valleix, whose classification has been followed by the principal later writers, into those of the lower part of the back of the neck, and lower cervical vertebræ; the axillary, in the hollow of the axilla; the scapular, corresponding to the angle of that bone; another, where the cutaneous branches of the circumflex nerve emerge through the deltoid muscle; another, where the musculo-spiral sends out its cutaneous branches, about three inches above the elbow; the median cephalic, at the bend of the elbow; the superior ulnar, at the internal posterior side of the elbow; the inferior ulnar, just above the annular ligament of the wrist; and lastly, the radial, at the lower external portion of the forearm. The fingers are frequently affected by severe and troublesome neuralgias.

The points at which pain is usually perceived in the next division of neuralgias, or dorso-intercostal, are the intervertebral foramen, near the sternum, and about the middle of the intercostal space, that is, intermediate between the first two. Pain is frequently developed in this latter point, immediately under the breast in women who have been debilitated by excessive lactation, by leucorrhœa, or other forms of uterine disorder; while neuralgia of the breast itself is by no means uncommon. In this form of the disease, the pain may be aggravated by inspiration, or by cough.

In dorso-lumbar neuralgias, the painful points will be found about the middle of the crista ilii, an abdominal point in the hypogastric region; another at the point of emergence of the spermatic cord, and another in the scrotum. Neuralgia of the penis is not uncommon.

Crural neuralgia, involving the superficial branches of the crural nerve, supplying the anterior portion of the thigh, is described by authors as very rare. I have seen but one case, about eight years ago.

The last group of external neuralgias to which I shall call your attention, is sciatica, one of the most frequent forms of all, and also one of the most obstinate and painful. This form is rarely found to exist in young persons, and emphatically belongs to the neuralgias of the period of degeneration, the subjects being old in constitution, if not in years; it is often associated with atheromatous deposits in the arteries, with arcus senilis, and with other evidences of retrograde metamorphoses of tissue.

In common with all forms of neuralgia, involving spinal nerves proper, sciatica manifests itself in the posterior, superficial, muscular, and cutaneous branches of the nerve implicated. The whole spinal column is rendered tender from this cause, and what is known as spinal irritation, frequently misleads those unfamiliar with it, into the error of suspecting grave lesion of the cord itself. The most prominent location of the pain of sciatica, is at the point where the nerve emerges from the pelvis; there is one also at the head of the fibula, in the course of the external popliteal nerve; others behind the external and internal malleoli; and still another, which I have not seen mentioned by any author, but which has occurred with great severity, under my own observation, upon the inner margin of the great toe. So much, gentlemen, for the topography of neuralgia of the external or superficial nerves. Something may be said, however, with regard to its complications.

These are, first, with motor nerves and their functions, manifesting themselves by convulsive or reflex movements, most apparent in the muscles of the face, in what is called spasmodic tic, or by Trousseau, tic epileptiforme, in the spasmodic contractions of the muscles of the face and of the eyelid, sometimes also of the extremities.

Another complication, and which seems to have only recently attracted the attention of observers, is with the trophic branches of the ganglionic nervous system, and is manifested by the occurrence of cutaneous eruptions; the most marked of which is herpes in its various forms, and of these more especially herpes zoster, or zona, commonly known as shingles, covering, with its vesicles, the area receiving its innervation from the neuralgic nerves, and, according to some authorities, marking the decline of the pain. This is, however, not always the case, the pain frequently persisting during the continuance of the eruption. I have a patient subject to attacks of herpes, periodically, who can foretell the approach of the eruption twenty-four hours in advance of its appearance, by the neuralgic pains which invariably precede it.

